# {4-[(Diphenyl­phosphino)methyl­amino]pyridinium-κ*P*}bis­(nitrato-κ*O*)silver(I)

**DOI:** 10.1107/S160053680903178X

**Published:** 2009-08-19

**Authors:** Jing Shang, Liu-Cheng Gui, Qing-Ling Ni, Min Zhong, Heng-Chi Lian

**Affiliations:** a School of Chemistry and Chemical Engineering, Guangxi Normal University, Guilin 541004, People’s Republic of China

## Abstract

In the title mononuclear complex, [Ag(C_18_H_18_N_2_P)(NO_3_)_2_], the metal centre is coordinated in a slightly distorted trigonal–planar geometry by the P atom of the phosphine ligand and the O atoms of the two monodentate nitrate anions. In the crystal structure, complex mol­ecules are connected by inter­molecular N—H⋯O hydrogen bonds, forming chains running parallel to the *b* axis.

## Related literature

For related structures, see: Song *et al.* (2002 ▶); Durran *et al.* (2006 ▶); Jiang *et al.* (2009 ▶); Wang *et al.* (2008 ▶). 
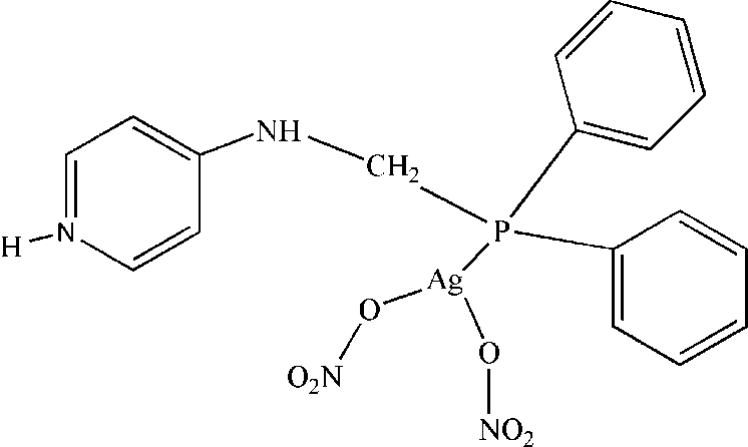


## Experimental

### 

#### Crystal data

[Ag(C_18_H_18_N_2_P)(NO_3_)_2_]
*M* *_r_* = 525.20
Triclinic, 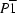

*a* = 7.9760 (9) Å
*b* = 9.6895 (11) Å
*c* = 14.1207 (16) Å
α = 86.170 (2)°
β = 89.170 (2)°
γ = 69.439 (2)°
*V* = 1019.5 (2) Å^3^
*Z* = 2
Mo *K*α radiation
μ = 1.11 mm^−1^
*T* = 173 K
0.47 × 0.33 × 0.16 mm

#### Data collection

Bruker SMART CCD area-detector diffractometer
Absorption correction: multi-scan (*SADABS*; Bruker, 1998 ▶) *T*_min_ = 0.699, *T*_max_ = 0.838
8623 measured reflections
4367 independent reflections
3802 reflections with *I* > 2σ(*I*)
*R*_int_ = 0.018

#### Refinement

*R*[*F* ^2^ > 2σ(*F* ^2^)] = 0.029
*wR*(*F* ^2^) = 0.109
*S* = 1.15
4367 reflections
271 parameters
H-atom parameters constrained
Δρ_max_ = 0.66 e Å^−3^
Δρ_min_ = −0.46 e Å^−3^

### 

Data collection: *SMART* (Bruker, 1998 ▶); cell refinement: *SAINT* (Bruker, 1998 ▶); data reduction: *SAINT*; program(s) used to solve structure: *SHELXS97* (Sheldrick, 2008 ▶); program(s) used to refine structure: *SHELXL97* (Sheldrick, 2008 ▶); molecular graphics: *SHELXTL* (Sheldrick, 2008 ▶); software used to prepare material for publication: *SHELXTL*.

## Supplementary Material

Crystal structure: contains datablocks I, global. DOI: 10.1107/S160053680903178X/rz2347sup1.cif

Structure factors: contains datablocks I. DOI: 10.1107/S160053680903178X/rz2347Isup2.hkl

Additional supplementary materials:  crystallographic information; 3D view; checkCIF report

## Figures and Tables

**Table 1:** Hydrogen-bond geometry (Å, °)

*D*—H⋯*A*	*D*—H	H⋯*A*	*D*⋯*A*	*D*—H⋯*A*
N1—H1*A*⋯O5^i^	0.88	1.93	2.807 (3)	172
N2—H2*A*⋯O4^ii^	0.88	2.16	3.013 (3)	162
N2—H2*A*⋯O5^ii^	0.88	2.47	3.075 (3)	127

Symmetry codes: (i) 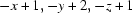; (ii) 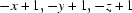.

**Fig. 1.**
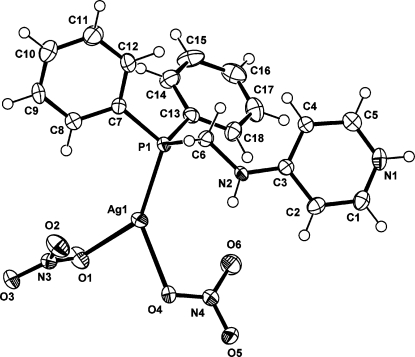
The molecular structure of title complex with the atom numbering scheme. Displacement ellipsoids are drawn at the 50% probability level.

**Fig. 2.**
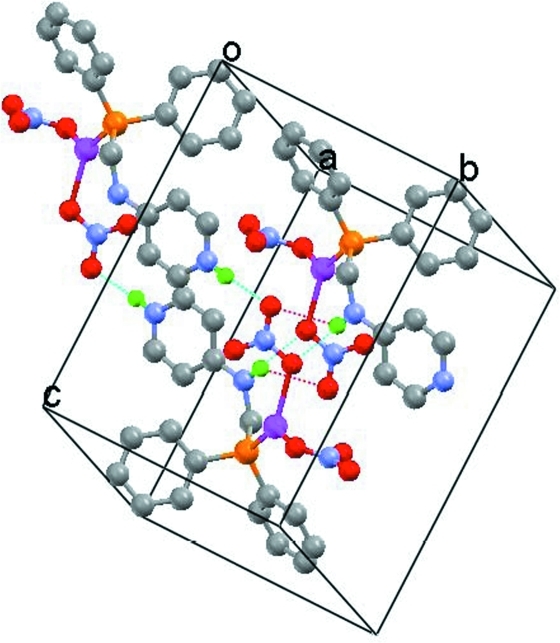
Partial packing diagram of the title compound, showing the intermlecular N—H···O hydrogen bonds as dashed lines. H atoms not involved in hydrogen bonding are omitted for clarity.

## supplementary crystallographic information

### Comment

Silver(I) metal complexes with pyridine-phosphine ligands which contain a soft Lewis base (P) and a hard Lewis acid (N) had been extensively studied so far (Song *et al.*, 2002; Durran *et al.*, 2006; Jiang *et al.*, 2009; Wang *et al.* 2008). Herein, we report the crystal structure of the title complex.

In the title complex (Fig. 1), the silver(I) metal centre adopts a slightly distorted trigonal-planar geometry provided by the P atom of the phosphine ligand and by the O atoms of two monodentate nitrate anions. The metal is displaced by 0.0629 (3) Å from the plane of the donor atoms. In the crystal structure, the complex molecules are linked by intermolecular N—H···O hydrogen bonds (Table 1, Fig. 2) to form chains running parallel to the *b* axis.

### Experimental

Silver nitrate (0.0168 g, 1 mmol) was dissolved in CH_3_CN (2 ml) and a solution of *N*-diphenylphosphinomethyl-4-aminopyridinium (0.0292 g, 1 mmol) in CH_3_OH (4 ml) was added with stirring for three hours at room temperature. Subsequent diethyl ether diffusion into the solution afforded colourless crystal of the title compound suitable for X-ray analysis.

### Refinement

All H atoms were located geometrically and treated as riding atoms, with N—H= 0.88 Å, C—H = 0.95–0.98 Å, and with *U*_iso_(H) = 1.2*U*_eq_(C, N).

### Crystal data

**Table:**

[Ag(C_18_H_18_N_2_P)(NO_3_)_2_]	*Z* = 2
*M**_r_* = 525.20	*F*(000) = 528
Triclinic, *P*1	*D*_x_ = 1.711 Mg m^−^^3^
Hall symbol: -P 1	Mo *K*α radiation, λ = 0.71073 Å
*a* = 7.9760 (9) Å	Cell parameters from 5807 reflections
*b* = 9.6895 (11) Å	θ = 2.7–27.0°
*c* = 14.1207 (16) Å	µ = 1.11 mm^−^^1^
α = 86.170 (2)°	*T* = 173 K
β = 89.170 (2)°	Block, colourless
γ = 69.439 (2)°	0.47 × 0.33 × 0.16 mm
*V* = 1019.5 (2) Å^3^	

### Data collection

**Table:**

Bruker SMART CCD area-detector diffractometer	4367 independent reflections
Radiation source: fine-focus sealed tube	3802 reflections with *I* > 2σ(*I*)
graphite	*R*_int_ = 0.018
ω scans	θ_max_ = 27.0°, θ_min_ = 2.6°
Absorption correction: multi-scan (*SADABS*; Bruker, 1998)	*h* = −10→9
*T*_min_ = 0.699, *T*_max_ = 0.838	*k* = −12→12
8623 measured reflections	*l* = −18→17

### Refinement

**Table:**

Refinement on *F*^2^	Primary atom site location: structure-invariant direct methods
Least-squares matrix: full	Secondary atom site location: difference Fourier map
*R*[*F*^2^ > 2σ(*F*^2^)] = 0.029	Hydrogen site location: inferred from neighbouring sites
*wR*(*F*^2^) = 0.109	H-atom parameters constrained
*S* = 1.15	*w* = 1/[σ^2^(*F*_o_^2^) + (0.0737*P*)^2^ + 0.0727*P*] where *P* = (*F*_o_^2^ + 2*F*_c_^2^)/3
4367 reflections	(Δ/σ)_max_ = 0.001
271 parameters	Δρ_max_ = 0.66 e Å^−^^3^
0 restraints	Δρ_min_ = −0.46 e Å^−^^3^

### Special details

**Table:**

Geometry. All e.s.d.'s (except the e.s.d. in the dihedral angle between two l.s. planes) are estimated using the full covariance matrix. The cell e.s.d.'s are taken into account individually in the estimation of e.s.d.'s in distances, angles and torsion angles; correlations between e.s.d.'s in cell parameters are only used when they are defined by crystal symmetry. An approximate (isotropic) treatment of cell e.s.d.'s is used for estimating e.s.d.'s involving l.s. planes.
Refinement. Refinement of *F*^2^ against ALL reflections. The weighted *R*-factor *wR* and goodness of fit *S* are based on *F*^2^, conventional *R*-factors *R* are based on *F*, with *F* set to zero for negative *F*^2^. The threshold expression of *F*^2^ > σ(*F*^2^) is used only for calculating *R*-factors(gt) *etc*. and is not relevant to the choice of reflections for refinement. *R*-factors based on *F*^2^ are statistically about twice as large as those based on *F*, and *R*- factors based on ALL data will be even larger.

### Fractional atomic coordinates and isotropic or equivalent isotropic displacement parameters (Å^2^)

**Table:**

	*x*	*y*	*z*	*U*_iso_*/*U*_eq_	
Ag1	0.46577 (3)	0.46190 (2)	0.297716 (16)	0.03069 (11)	
P1	0.20209 (10)	0.64942 (8)	0.24168 (5)	0.02155 (16)	
O1	0.6827 (3)	0.2456 (3)	0.24276 (18)	0.0391 (5)	
O2	0.4738 (3)	0.1861 (3)	0.31211 (19)	0.0429 (6)	
O3	0.6975 (3)	0.0183 (2)	0.25015 (18)	0.0428 (6)	
O4	0.6546 (3)	0.4154 (2)	0.43169 (15)	0.0311 (5)	
O5	0.7902 (3)	0.5171 (2)	0.52073 (17)	0.0381 (6)	
O6	0.6328 (4)	0.6441 (3)	0.39968 (18)	0.0481 (7)	
N1	0.1753 (3)	1.2090 (3)	0.45460 (18)	0.0283 (5)	
H1A	0.1774	1.2963	0.4667	0.034*	
N2	0.1655 (3)	0.7994 (2)	0.40482 (16)	0.0219 (5)	
H2A	0.2357	0.7271	0.4428	0.026*	
N3	0.6180 (4)	0.1481 (3)	0.26837 (18)	0.0288 (5)	
N4	0.6919 (3)	0.5284 (3)	0.44974 (17)	0.0265 (5)	
C1	0.2731 (4)	1.0885 (3)	0.5089 (2)	0.0269 (6)	
H1B	0.3442	1.0991	0.5594	0.032*	
C2	0.2720 (4)	0.9528 (3)	0.4930 (2)	0.0256 (6)	
H2B	0.3420	0.8693	0.5321	0.031*	
C3	0.1658 (3)	0.9348 (3)	0.41738 (19)	0.0204 (5)	
C4	0.0664 (4)	1.0632 (3)	0.3620 (2)	0.0231 (6)	
H4A	−0.0059	1.0567	0.3108	0.028*	
C5	0.0740 (4)	1.1971 (3)	0.3818 (2)	0.0284 (6)	
H5A	0.0070	1.2831	0.3438	0.034*	
C6	0.0617 (4)	0.7590 (3)	0.33523 (19)	0.0230 (5)	
H6A	−0.0272	0.8500	0.3051	0.028*	
H6B	−0.0049	0.7010	0.3680	0.028*	
C7	0.0424 (4)	0.5829 (3)	0.18720 (19)	0.0229 (5)	
C8	0.0942 (4)	0.4339 (3)	0.1700 (2)	0.0325 (7)	
H8A	0.2135	0.3697	0.1835	0.039*	
C9	−0.0290 (5)	0.3790 (3)	0.1330 (3)	0.0394 (8)	
H9A	0.0068	0.2772	0.1217	0.047*	
C10	−0.1997 (5)	0.4697 (4)	0.1129 (2)	0.0375 (8)	
H10A	−0.2831	0.4310	0.0884	0.045*	
C11	−0.2522 (4)	0.6186 (4)	0.1281 (2)	0.0378 (8)	
H11A	−0.3711	0.6822	0.1129	0.045*	
C12	−0.1322 (4)	0.6748 (3)	0.1652 (2)	0.0335 (7)	
H12A	−0.1692	0.7770	0.1758	0.040*	
C13	0.2342 (4)	0.7867 (3)	0.15637 (19)	0.0227 (5)	
C14	0.1854 (4)	0.7949 (4)	0.0613 (2)	0.0339 (7)	
H14A	0.1303	0.7297	0.0404	0.041*	
C15	0.2167 (5)	0.8975 (4)	−0.0029 (2)	0.0424 (8)	
H15A	0.1858	0.9011	−0.0680	0.051*	
C16	0.2934 (5)	0.9955 (4)	0.0282 (3)	0.0414 (8)	
H16A	0.3104	1.0689	−0.0150	0.050*	
C17	0.3444 (5)	0.9857 (4)	0.1215 (3)	0.0378 (8)	
H17A	0.3977	1.0519	0.1425	0.045*	
C18	0.3189 (4)	0.8801 (3)	0.1855 (2)	0.0285 (6)	
H18A	0.3593	0.8716	0.2493	0.034*	

### Atomic displacement parameters (Å^2^)

**Table:**

	*U*^11^	*U*^22^	*U*^33^	*U*^12^	*U*^13^	*U*^23^
Ag1	0.03087 (16)	0.02167 (15)	0.03252 (16)	0.00010 (10)	−0.00920 (10)	−0.00312 (9)
P1	0.0243 (4)	0.0165 (3)	0.0215 (3)	−0.0036 (3)	−0.0032 (3)	−0.0036 (3)
O1	0.0364 (13)	0.0254 (12)	0.0523 (15)	−0.0074 (10)	0.0050 (11)	−0.0014 (10)
O2	0.0350 (13)	0.0333 (13)	0.0535 (15)	−0.0049 (10)	0.0092 (11)	0.0023 (11)
O3	0.0476 (14)	0.0205 (11)	0.0509 (15)	0.0013 (10)	−0.0151 (11)	−0.0095 (10)
O4	0.0377 (12)	0.0186 (10)	0.0362 (12)	−0.0080 (9)	−0.0105 (9)	−0.0035 (8)
O5	0.0479 (14)	0.0260 (12)	0.0433 (13)	−0.0169 (11)	−0.0224 (11)	0.0037 (10)
O6	0.0708 (18)	0.0321 (13)	0.0454 (15)	−0.0250 (13)	−0.0217 (13)	0.0142 (11)
N1	0.0319 (13)	0.0239 (13)	0.0343 (14)	−0.0151 (11)	0.0092 (11)	−0.0096 (10)
N2	0.0283 (12)	0.0136 (11)	0.0207 (11)	−0.0034 (9)	−0.0046 (9)	−0.0004 (8)
N3	0.0320 (14)	0.0225 (13)	0.0275 (13)	−0.0035 (11)	−0.0083 (10)	−0.0036 (10)
N4	0.0292 (13)	0.0214 (12)	0.0271 (12)	−0.0068 (10)	−0.0038 (10)	0.0000 (10)
C1	0.0248 (14)	0.0313 (16)	0.0276 (15)	−0.0123 (12)	0.0047 (11)	−0.0092 (12)
C2	0.0234 (14)	0.0331 (16)	0.0205 (13)	−0.0096 (12)	0.0016 (10)	−0.0042 (11)
C3	0.0171 (12)	0.0223 (14)	0.0205 (13)	−0.0049 (10)	0.0035 (10)	−0.0043 (10)
C4	0.0230 (13)	0.0214 (14)	0.0240 (14)	−0.0064 (11)	−0.0002 (10)	−0.0025 (11)
C5	0.0286 (15)	0.0275 (16)	0.0277 (15)	−0.0085 (12)	0.0053 (11)	−0.0001 (11)
C6	0.0243 (13)	0.0240 (14)	0.0202 (13)	−0.0074 (11)	−0.0008 (10)	−0.0039 (10)
C7	0.0267 (14)	0.0198 (13)	0.0229 (13)	−0.0086 (11)	−0.0025 (11)	−0.0024 (10)
C8	0.0340 (17)	0.0243 (16)	0.0362 (17)	−0.0058 (13)	−0.0082 (13)	−0.0045 (12)
C9	0.053 (2)	0.0170 (15)	0.049 (2)	−0.0121 (15)	−0.0120 (16)	−0.0056 (13)
C10	0.0388 (18)	0.0405 (19)	0.0408 (18)	−0.0222 (16)	−0.0018 (14)	−0.0082 (14)
C11	0.0276 (16)	0.043 (2)	0.0406 (19)	−0.0075 (14)	0.0001 (13)	−0.0133 (15)
C12	0.0316 (16)	0.0225 (16)	0.0429 (18)	−0.0033 (13)	−0.0039 (13)	−0.0111 (13)
C13	0.0206 (13)	0.0209 (13)	0.0233 (13)	−0.0030 (11)	0.0012 (10)	−0.0025 (10)
C14	0.0360 (17)	0.0389 (18)	0.0293 (16)	−0.0166 (15)	−0.0054 (13)	0.0011 (13)
C15	0.0381 (19)	0.063 (2)	0.0269 (16)	−0.0213 (18)	−0.0099 (13)	0.0133 (15)
C16	0.0333 (17)	0.0385 (19)	0.049 (2)	−0.0107 (15)	0.0033 (15)	0.0135 (15)
C17	0.0393 (19)	0.0294 (17)	0.048 (2)	−0.0156 (15)	0.0118 (15)	−0.0065 (14)
C18	0.0282 (15)	0.0315 (16)	0.0276 (15)	−0.0118 (13)	0.0031 (12)	−0.0081 (12)

### Geometric parameters (Å, °)

**Table:**

Ag1—P1	2.3500 (8)	C5—H5A	0.9500
Ag1—O4	2.354 (2)	C6—H6A	0.9900
Ag1—O1	2.370 (2)	C6—H6B	0.9900
P1—C7	1.817 (3)	C7—C12	1.391 (4)
P1—C13	1.817 (3)	C7—C8	1.393 (4)
P1—C6	1.854 (3)	C8—C9	1.393 (4)
O1—N3	1.257 (3)	C8—H8A	0.9500
O2—N3	1.246 (3)	C9—C10	1.358 (5)
O3—N3	1.236 (3)	C9—H9A	0.9500
O4—N4	1.273 (3)	C10—C11	1.385 (5)
O5—N4	1.256 (3)	C10—H10A	0.9500
O6—N4	1.229 (3)	C11—C12	1.381 (5)
N1—C1	1.349 (4)	C11—H11A	0.9500
N1—C5	1.352 (4)	C12—H12A	0.9500
N1—H1A	0.8800	C13—C18	1.390 (4)
N2—C3	1.336 (3)	C13—C14	1.393 (4)
N2—C6	1.452 (3)	C14—C15	1.387 (5)
N2—H2A	0.8800	C14—H14A	0.9500
C1—C2	1.351 (4)	C15—C16	1.394 (5)
C1—H1B	0.9500	C15—H15A	0.9500
C2—C3	1.430 (4)	C16—C17	1.371 (5)
C2—H2B	0.9500	C16—H16A	0.9500
C3—C4	1.408 (4)	C17—C18	1.386 (4)
C4—C5	1.368 (4)	C17—H17A	0.9500
C4—H4A	0.9500	C18—H18A	0.9500
			
P1—Ag1—O4	136.46 (5)	P1—C6—H6A	109.0
P1—Ag1—O1	138.31 (7)	N2—C6—H6B	109.0
O4—Ag1—O1	84.99 (8)	P1—C6—H6B	109.0
C7—P1—C13	105.94 (13)	H6A—C6—H6B	107.8
C7—P1—C6	100.58 (13)	C12—C7—C8	118.8 (3)
C13—P1—C6	104.39 (13)	C12—C7—P1	122.2 (2)
C7—P1—Ag1	114.33 (10)	C8—C7—P1	119.0 (2)
C13—P1—Ag1	115.36 (9)	C7—C8—C9	120.0 (3)
C6—P1—Ag1	114.65 (9)	C7—C8—H8A	120.0
N3—O1—Ag1	102.13 (18)	C9—C8—H8A	120.0
N4—O4—Ag1	111.82 (16)	C10—C9—C8	120.7 (3)
C1—N1—C5	120.7 (3)	C10—C9—H9A	119.7
C1—N1—H1A	119.7	C8—C9—H9A	119.7
C5—N1—H1A	119.7	C9—C10—C11	120.0 (3)
C3—N2—C6	126.9 (2)	C9—C10—H10A	120.0
C3—N2—H2A	116.5	C11—C10—H10A	120.0
C6—N2—H2A	116.5	C12—C11—C10	120.2 (3)
O3—N3—O2	121.7 (3)	C12—C11—H11A	119.9
O3—N3—O1	120.0 (3)	C10—C11—H11A	119.9
O2—N3—O1	118.3 (3)	C11—C12—C7	120.4 (3)
O6—N4—O5	121.6 (2)	C11—C12—H12A	119.8
O6—N4—O4	121.0 (2)	C7—C12—H12A	119.8
O5—N4—O4	117.5 (2)	C18—C13—C14	119.1 (3)
N1—C1—C2	121.3 (3)	C18—C13—P1	119.2 (2)
N1—C1—H1B	119.3	C14—C13—P1	121.5 (2)
C2—C1—H1B	119.3	C15—C14—C13	120.4 (3)
C1—C2—C3	120.1 (3)	C15—C14—H14A	119.8
C1—C2—H2B	120.0	C13—C14—H14A	119.8
C3—C2—H2B	120.0	C14—C15—C16	119.8 (3)
N2—C3—C4	124.7 (2)	C14—C15—H15A	120.1
N2—C3—C2	118.4 (3)	C16—C15—H15A	120.1
C4—C3—C2	116.9 (3)	C17—C16—C15	119.7 (3)
C5—C4—C3	120.0 (3)	C17—C16—H16A	120.1
C5—C4—H4A	120.0	C15—C16—H16A	120.1
C3—C4—H4A	120.0	C16—C17—C18	120.8 (3)
N1—C5—C4	121.0 (3)	C16—C17—H17A	119.6
N1—C5—H5A	119.5	C18—C17—H17A	119.6
C4—C5—H5A	119.5	C17—C18—C13	120.1 (3)
N2—C6—P1	112.86 (19)	C17—C18—H18A	120.0
N2—C6—H6A	109.0	C13—C18—H18A	120.0
			
O4—Ag1—P1—C7	−139.96 (13)	C13—P1—C7—C12	−63.3 (3)
O1—Ag1—P1—C7	47.87 (14)	C6—P1—C7—C12	45.2 (3)
O4—Ag1—P1—C13	96.81 (13)	Ag1—P1—C7—C12	168.5 (2)
O1—Ag1—P1—C13	−75.36 (13)	C13—P1—C7—C8	119.1 (2)
O4—Ag1—P1—C6	−24.55 (14)	C6—P1—C7—C8	−132.4 (2)
O1—Ag1—P1—C6	163.29 (13)	Ag1—P1—C7—C8	−9.1 (3)
P1—Ag1—O1—N3	−89.92 (19)	C12—C7—C8—C9	−1.1 (5)
O4—Ag1—O1—N3	95.49 (19)	P1—C7—C8—C9	176.6 (3)
P1—Ag1—O4—N4	−46.8 (2)	C7—C8—C9—C10	0.3 (5)
O1—Ag1—O4—N4	128.01 (19)	C8—C9—C10—C11	0.8 (6)
Ag1—O1—N3—O3	179.4 (2)	C9—C10—C11—C12	−1.1 (5)
Ag1—O1—N3—O2	−0.7 (3)	C10—C11—C12—C7	0.3 (5)
Ag1—O4—N4—O6	−0.5 (4)	C8—C7—C12—C11	0.7 (5)
Ag1—O4—N4—O5	179.1 (2)	P1—C7—C12—C11	−176.9 (3)
C5—N1—C1—C2	−0.5 (4)	C7—P1—C13—C18	166.2 (2)
N1—C1—C2—C3	0.0 (4)	C6—P1—C13—C18	60.5 (3)
C6—N2—C3—C4	−1.9 (4)	Ag1—P1—C13—C18	−66.2 (2)
C6—N2—C3—C2	177.3 (2)	C7—P1—C13—C14	−17.5 (3)
C1—C2—C3—N2	−178.8 (3)	C6—P1—C13—C14	−123.2 (3)
C1—C2—C3—C4	0.4 (4)	Ag1—P1—C13—C14	110.0 (2)
N2—C3—C4—C5	178.9 (3)	C18—C13—C14—C15	−1.7 (5)
C2—C3—C4—C5	−0.2 (4)	P1—C13—C14—C15	−177.9 (3)
C1—N1—C5—C4	0.7 (4)	C13—C14—C15—C16	−1.6 (5)
C3—C4—C5—N1	−0.3 (4)	C14—C15—C16—C17	2.7 (6)
C3—N2—C6—P1	111.1 (3)	C15—C16—C17—C18	−0.6 (5)
C7—P1—C6—N2	167.0 (2)	C16—C17—C18—C13	−2.7 (5)
C13—P1—C6—N2	−83.4 (2)	C14—C13—C18—C17	3.8 (4)
Ag1—P1—C6—N2	43.8 (2)	P1—C13—C18—C17	−179.9 (2)

### Hydrogen-bond geometry (Å, °)

**Table:**

*D*—H···*A*	*D*—H	H···*A*	*D*···*A*	*D*—H···*A*
N1—H1A···O5^i^	0.88	1.93	2.807 (3)	172
N2—H2A···O4^ii^	0.88	2.16	3.013 (3)	162
N2—H2A···O5^ii^	0.88	2.47	3.075 (3)	127

Symmetry codes: (i) −*x*+1, −*y*+2, −*z*+1; (ii) −*x*+1, −*y*+1, −*z*+1.
